# Supplementary material for: Are carbon emissions trading and green financial instruments synergistic? -Comprehensive quantitative research based on content analysis
Source: PLoS One. 2024 Mar 7;19(3):e0298601. doi: 10.1371/journal.pone.0298601 (PMC10919601; doi:10.1371/journal.pone.0298601)
Supplement: S1 Data — (ZIP) [file pone.0298601.s001.zip › Data/地级市Z_F工作报告.zip!/░▓╗╒/╬▀║■╩╨/└┤╫╘╣1⁄2╓┌║┼í╛┬φ┐╦╩2╛▌═°í┐_2019.docx]

各位代表：

现在，我代表市人民政府，向大会报告政府工作，请予审议，并请市政协各位委员提出意见。

一、2018年工作回顾

过去一年，全市人民在中央、省委省政府及市委的坚强领导下，坚持以习近平新时代中国特色社会主义思想为指导，坚持稳中求进工作总基调，以供给侧结构性改革为主线，以五大发展行动计划为总抓手，着力打好防范化解重大风险、精准脱贫、污染防治三大攻坚战，落实高质量发展要求，较好完成了市十六届人大一次会议确定的年度目标任务。

初步核算，全市生产总值3278.5亿元，比上年增长8.4%；财政收入603亿元，增长8%；居民人均可支配收入31445元，增长9.9%；城镇新增就业9.2万人，城镇登记失业率控制在2.8%；单位生产总值能耗下降6.5%；生态环境质量稳中向好。我市获批建设国家创新型城市，成为国家森林城市、水生态文明城市、质量魅力城市、消费品工业“三品”示范城市，入选中国改革开放40周年发展最成功的40座城市、数字经济百强城市。

一年来，我们主要做了以下六个方面的工作。

（一）注重提质增效，发展质量进一步提高。

激发民营经济活力。出台了促进经济高质量发展、改进企业服务优化营商环境、大力支持民营经济发展35条等政策措施，修订扶持产业发展“1+5+6”政策体系。开展全国质量强市示范城市创建工作。深化“一企一组一策”帮扶机制，深入开展“四送一服”双千工程，兑付支持企业发展奖补资金34亿元，减轻企业税费负担121亿元。25家企业开展电力直接交易。市场主体27万户，其中企业数量8万家，增长19%。39家企业入选安徽省百强民营企业，居全省第一。

增强金融服务实体经济能力。新增伯特利首发上市，信义能源获准在香港上市，6家企业在新三板挂牌，完成直接融资369亿元。新增政银担、税融通、续贷过桥业务132亿元。金融机构本外币存、贷款余额分别比年初增加488亿元、202亿元。

释放投资推动发展动力。落实“四督四保”制度要求，建立重点项目建设六项工作机制。强化重点项目动态管控，新开工亿元以上项目514个。出台了进一步激发民间有效投资活力、促进经济持续健康发展的实施意见，民间投资增长17.3%。

提升创新要素推动发展效力。组建了市产业创新中心。出台了支持战略性新兴产业发展人才专项政策，引进高层次科技人才团队76个。新批国家地方联合工程研究中心、国家级企业技术中心2家，国家级博士后科研工作站2家，省级研发机构21家，建成国家工业机器人产品质量监督检验中心（安徽），中科院上海光机（芜湖）激光应用示范中心投入运营。西电芜湖研究院正式招生。举办大院大所合作暨科技成果对接会、全国机器人专利等系列创新创业大赛、“我有嘉宾”独角兽峰会，万人有效发明专利拥有量突破31件。芜湖东旭公司参与项目荣获国家科技进步一等奖。新增高新技术企业130家。新增省级青年创业园1家。

（二）注重补齐短板，攻坚成效进一步凸显。

坚决打赢防范化解重大风险攻坚战。严格规范政府债务管理、政府性投资项目。推进融资平台市场化转型。新增政府债券16.5亿元，完成政府存量债务置换152.3亿元，年节约资金成本7亿元。制定了政府隐性债务化解方案，排查处置网贷、非法集资等风险，组织化解电缆行业联保互保风险和少数企业债务风险。

坚决打赢精准脱贫攻坚战。安排财政专项扶贫资金3.5亿元，开展“重补促”专项整改行动、精准扶贫“百日攻坚”行动。实现10841人脱贫。启动无扶贫开发任务县区困难群体帮扶。完成农村危房改造5327户，解决农村8476人饮水不安全问题。

坚决打赢污染防治攻坚战。贯彻中央和省生态环保督察及省委巡视整改要求，扎实做好反馈问题整改。完成大气污染整治项目1168个。推进水污染防治行动计划，建立地表水断面生态补偿机制，国家考核水质断面综合达标率100%。大龙湾等4座污水处理厂新建及提标改造工程开工。基本完成城东污水管网整治。完成24条黑臭水体治理。加强固体废物排查整治，及时实施无害化处置。开展自然保护区监督检查专项行动。推进第二次污染源普查。餐厨废弃物资源化利用和无害化处理试点城市通过验收。完成造林5.3万亩、森林长廊60公里，新增绿地155.6万平方米。完成16家矿山地质环境恢复治理，建成20家市级绿色矿山。芜湖县成为国家生态文明建设示范县。

（三）注重释放活力，改革步伐进一步加快。

全面深化改革。中小企业信用贷发放16亿元。荣获守信激励创新奖。在全省首创优化营商环境18条措施、事中事后监管制度模板，企业开办时间压缩至3个工作日。盘活低效工业用地4400亩，处置闲置土地2100亩。改革驻点招商模式。“傻子瓜子”创始人年广九入选“改革开放40年百名杰出民营企业家”。

全面扩大开放。参与长三角区域大通关建设，实现“单一窗口”报关报检全覆盖。省港口运营集团落户芜湖。口岸进出口货运量超500万吨，集装箱吞吐量超80万标箱。三山港对外开放获批筹建。复制推广自贸区改革试点经验，成为全国供应链创新与应用试点城市。进出口总额68.8亿美元。共同发布《G60科创走廊松江宣言》，在上海虹桥商务区建立城市展厅。出台推进中外合作园区发展政策规定。实施外商投资企业商务备案与工商登记“一口办理”，开展“委托受理、远程核准”试点。新批准外商投资企业26家，实际利用外资29.2亿美元。与德国合作推广双元制职业教育模式，深化经济产业合作。

全面推进“互联网+政务服务”。智慧城市协同创新中心建成，易政网全面启用。优化政府权责清单、公共服务清单、行政权力中介服务清单，开展“减证便民”，政务服务事项申请材料精简57%。实施“证照分离”改革试点。政府大数据库建设全国领先。在全国首创网约车巡游车市场准入同门槛、产业联盟同治理，网约车治理改革获中国法治政府奖。启动国家政务服务标准化项目试点和标准化体系建设，建成全省首个网上办事智能自助大厅。实施G60科创走廊“一网通办”试点。

（四）注重扶持实体，产业结构进一步优化。

传统产业加速转型。谋划储备重点技改投资项目219个，实施亿元以上技改项目260个。汽车、材料、电器、电缆产业增加值增长6.9%。奇瑞集团销售再创新高，年营业收入跨越千亿元台阶。海螺获评省政府质量奖。美的空调产能进一步扩大。启动企业兼并重组项目20个。

新动能加速培育。战略性新兴产业增加值增长11.8%。聚焦培育机器人及智能装备、新能源汽车、航空等战略性新兴产业。新型显示、海洋工程装备2个重大工程，智能物流装备、高端医疗装备、燃料电池系统3个重大专项，入选第三批省重大新兴产业工程和专项。埃夫特六关节机器人销量稳居全国第一。奇瑞新能源汽车细分市场占有率全国第二。中联重机智能烘干系统、甘蔗收割机位列全国产销量首位。中电科钻石飞机CA20型样机首飞成功。中车浦镇庞巴迪跨座式单轨及APM车辆实现产品交付。开展市“十三五”军民融合深度发展规划、省级军民融合产业示范基地建设方案编制。

服务业比重加速提升。服务业比重达43.8%。现代物流、金融、文化创意、旅游和服务外包等重点服务业企业实现营业收入增长10.2%。三只松鼠电子商务区获批省级现代服务业集聚区。快递业务量1.4亿件。成功举办第八届科博会、第五届中国汽车（房车）露营大会、安徽省第五届动漫大赛暨首届乡村文创大赛。社会消费品零售总额增长12.2%，其中网上零售额增长41%。全年接待游客增长21%。

现代农业加速壮大。新增家庭农场593家、农民专业合作社517家。“三品一标”有效认证108个，“芜湖大米”获得农产品地理标志认证。建设高标准农田11万亩，整理复垦开发补充耕地、增减挂钩及工矿废弃地复垦1.3万亩。完成“大棚房”问题专项清理整治。繁昌县完成农村产权制度改革全国试点县任务。

（五）注重优化布局，城乡面貌进一步改善。

立足自然规律开展城乡规划。围绕全面打造水清岸绿产业优美丽长江（芜湖）经济带的要求，修订了城市总体规划及海绵城市建设、城市供水、慢行系统、生态网络、湿地保护、龙窝湖等规划，推进市县“多规合一”，建立统一的空间规划数据库。

立足开放共享打造城乡交通。中山桥改建工程、芜湖火车站改扩建工程、黄山东路东延建成。弋江路快速化改造全线通车。商合杭高铁、芜宣机场、轨道交通1号线和2号线一期等项目稳步推进。沪武高速无为段、八里湾大桥、皖赣铁路湾沚南站等工程开工。农村道路畅通工程完成396公里。

立足规范精细管理美丽家园。推进“两治三改”，整治违法建设9.5万平方米、违法用地24宗，城镇棚户区改造新开工2258套，城中村改造项目新开工10432套，43个老旧小区改造完成。实施加装电梯试点。新改建城区厕所143座，改造农户厕所7.7万座。出台了互联网租赁自行车、户外广告设置、市区餐厨废弃物管理办法。新增分配公租房6032套，完成租赁补贴3683户。房地产市场总体平稳。殷港艺创小镇入围全国最美特色小镇50强。

（六）注重共建共享，民生福祉进一步提升。

以实干稳就业。发放稳岗就业补贴5893万元。完善技工强市的配套政策，评定市级技能大师工作室13家，新增技工学校1家。获批飞机维修、机电一体化2个世界技能大赛中国集训基地。开展“百名仲裁员服务千户企业”活动。

以实措强保障。实施全民参保登记计划，启动工伤保险“同舟计划”二期，城镇职工五项保险参保301万人次，城乡居民养老保险参保158万人、城镇居民医疗保险参保105万人。城乡居民养老保险基础养老金最高提升到每月160元。机关事业单位养老保险制度改革稳步推进。在全省率先完成低保标准城乡统筹，保障低保对象11.2万人，直接救助困难群众看病8.4万人次，资助困难群众参合参保17.4万人。发放住房公积金贷款支持9860户家庭购房安居。发放建档立卡贫困家庭学生资助金2306万元。

以实绩惠民生。投入110亿元，实施33项民生工程。启动第三期学前教育行动计划，新建公办幼儿园5个。推进义务教育优质均衡发展，消除66人以上超大班额。芜湖县、弋江区试点“幼升小”网上报名。我市获评“第一批教育信息化试点”区域性优秀单位。推进现代学徒制试点，成立华师大长三角职教研究院专家工作室。中航油芜湖培训学院开办。市中医院儿童治疗中心、市四院和皖南康复医院住院楼建成使用。智慧医院、网上预约挂号全面上线。建立2个医学院士工作站、19个医疗联合体。推行公立医院编制周转池制度改革。全面两孩政策稳步实施。精神卫生综合管理经验在全国推广。在全省率先推进媒体融合，成立市级传媒集团。完成国家文化消费试点城市工作。不动产登记在全省率先实现一小时办结。“城市令”APP提供惠民服务218项。举办了第七届中国·芜湖刘开渠奖国际雕塑大展，芜湖雕塑艺术馆开馆。芜湖铁画锻制技艺入选第一批国家传统工艺振兴目录。

以压责保稳定。全国社区治理和服务创新实验区工作通过验收。完成全市931个村（居）民委员会换届选举。退役军人就业安置、困难帮扶、优待抚恤等工作扎实推进。加强食品药品安全事中事后监管。成功处置突发传染病疫情，妥善处理长春长生疫苗事件、幼儿园食品安全问题。加强非洲猪瘟疫情防控。稳步推进全国消费投诉公示试点。深入开展“铸安”行动，安全生产形势稳定向好。市区11万套安置房完成初始登记。实施法律援助案件4674件。积极推进“扫黑除恶”专项斗争，公共安全视频监控基本实现重点区域全覆盖，社会治安总体平稳。支持工会、共青团、妇联等群团组织发挥作用。民族、宗教、外事、侨务、对台、人防、老龄、残疾人、档案等工作进一步发展。

以整改促提升。去年，政府系统认真做好省委巡视反馈意见整改，开展了“讲忠诚、严纪律、立政德”专题警示教育，肃清“陈、杨”遗毒遗风遗痕，净化优化政治生态。出台了《市政府工作规则》《市政府常务会议议事规则》，不断补齐制度短板。规范了政府合同签订程序，积极防范政府签约履约法律风险。市政府党组理论学习进一步加强，开展各类专题学习13次。办理人大代表建议183件，政协提案343件。3条服务热线整合纳入“12345政府服务直通车”，政府服务热线办复率100%。开展形式主义、官僚主义问题大调研大排查大整改活动，巩固深化作风建设成果。加强审计监督，完成对47位领导干部经济责任审计。

各位代表！

2018年是经济运行稳中有变、变中有忧并受“陈、杨”遗毒遗风遗痕影响不平稳的一年，是幼儿园食品安全、生态环境问题集中爆发不平静的一年，是在艰难条件下基本满足“六稳”工作要求不平凡的一年。这些成绩的取得，是中央、省委省政府和市委正确领导的结果，是市人大、市政协监督支持的结果，是全市人民拼搏奉献的结果。在这里，我谨代表芜湖市人民政府，向全市人民，向全体人大代表和政协委员，向各民主党派、各人民团体和各界人士，向驻芜部队，向关心支持芜湖发展的上级部门、兄弟市和海内外友好人士，表示崇高的敬意和诚挚的感谢！

我们也清醒认识到，芜湖发展中还存在很多不足：部分民营企业生产经营困难，营商环境有待改善；经济发展质量不够高，新旧动能转换和产业升级步伐有待加快；公共服务和基础设施存在短板，上学、看病、养老、出行等方面与群众期盼有差距；环境保护任务艰巨，空气质量、黑臭水体治理等有待改善；城乡之间、区域之间发展不平衡，部分农村人口仍未脱贫；作风建设有待加强，怕作为、慢作为、不担当和腐败现象仍有发生。对于这些问题，我们要采取有效措施，努力加以解决。

二、2019年主要工作

2019年是新中国成立70周年，是全面建成小康社会、实现第一个百年奋斗目标的关键之年。放眼全球，我们正面临百年未有之大变局，外部环境复杂严峻。芜湖单纯经济指标“一俊遮百丑”的时代效应必须打破，贯彻新时代新思想、重民生补短板的发展转型期已经到来。同时更应看到，新时代是中华民族大发展大作为的时代，我国发展仍处于并将长期处于重要战略机遇期。我们要积极对接全省港口整合、长江经济带发展、社会和谐稳定、长三角更高质量一体化、省委巡视整改和环保问题整治、“一带一路”等发展战略和机遇，抓住主要矛盾，有针对性地加以解决，在“巩固、增强、提升、畅通”八个字上下功夫，加快建设五大发展新芜湖。

今年政府工作的总体要求是：以习近平新时代中国特色社会主义思想为指导，统筹推进“五位一体”总体布局，协调推进“四个全面”战略布局，落实中央、省委省政府和市委部署，坚持稳中求进工作总基调，坚持新发展理念，坚持推动高质量发展，坚持以供给侧结构性改革为主线，坚持深化市场化改革、扩大高水平开放，加快建设现代化科教人才体系、产业体系、城乡体系、治理体系，全力打好三大攻坚战，统筹推进稳增长、促改革、调结构、惠民生、防风险工作，进一步做好“六稳”工作，增强人民群众获得感、幸福感、安全感，保持经济持续健康发展和社会大局稳定，为决胜全面建成小康社会打下决定性基础，打造现代化创新之城。

今年经济社会发展主要预期目标是：地区生产总值增长8%左右，财政收入增长、居民收入增长与经济增长同步，城镇新增就业7万人，城镇登记失业率控制在4.5%以内，节能减排完成省政府下达任务。

重点办“实”三件事：一要从实抓好发展的事。坚定不移推动高质量发展，坚持“两个毫不动摇”，促进多种所有制经济共同发展，巩固“三去一降一补”成果，切实做好“六稳”工作。二要扎实办好群众的事。坚定不移站稳人民立场，深入推进贴近群众“六走进”行动，持续深化“互联网+政务服务”和“最多跑一次”改革，不断满足人民群众对美好生活的向往，打造宜居宜业宜创宜游宜学宜养城市。三要切实管好自己的事。狠抓政风建设，彻底肃清“陈、杨”遗毒遗风遗痕，确保上级各项决策部署落地生根，提升政府公信力和执行力。

为实现上述目标，重点抓好以下七个方面工作：

（一）奋力抓生态，恢复自然机能。

良好的生态环境就是最大的生产力、最大的竞争力。我们要深入践行习近平生态文明思想，把经济景气转化为文明景象，把经济生态转化为社会生态，不断满足人民群众对美好生态的向往。

抓好共治共享大行动。扎实推进蓝天、碧水、净土三大保卫战。实施“三减三增七补齐”绿色行动，削减污染物排放总量、削减煤炭消费总量、削减落后产能规模；增林地、增绿地、增湿地；补齐污水处理短板、补齐黑臭水体治理短板、补齐工业园区生态环境治理短板、补齐长江生态保护修复短板、补齐农村面源污染治理短板、补齐农村人居环境短板、补齐生态保护地和固体废物监管短板，扎实推进水污染治理、水生态修复、水资源保护“三水共治”，全面提升生态环保治理能力、全面提升生态环境水平。纵深推进河长制、湖长制、林长制，落实打造水清岸绿产业优美丽长江（芜湖）经济带的各项举措，抓好中央生态环保督察“回头看”、省生态环保督察反馈问题整改。深化生态环保管理体制改革。

抓好自然生态大环境。以农村厕所、垃圾、污水治理“三大革命”为抓手，整治农村人居环境。全面推进生活垃圾分类。严守生态保护红线，提升生态系统质量和稳定性。落实最严格的生态环境保护制度，持续推进矿山地质环境恢复治理，开展绿色矿山创建，严格管控生态功能区、自然保护区、矿产资源禁采区、长江及重要河道两侧可视范围等区域矿业权，严厉打击非法偷盗采矿产资源行为。实施生态环境保护与修复，构建生态廊道和生物多样性保护网络。提升耕地保护水平，促进节约集约用地，深化土地利用方式转变。全面开展第三次全国国土资源调查，准确划定生态保护红线、永久基本农田、城镇开发边界三条控制线。

抓好节能降耗大统筹。加强节能控煤目标预警监测，严控高能耗高污染项目准入，推进能耗“双控”目标落实。推进电网、天然气等基础设施项目建设，多渠道筹措气源，保障天然气供应。实施新一轮农网升级改造，积极提高农村电力供应能力。

（二）发力转方式，提升发展势能。

“外因是变化的条件，内因是变化的根据”。我们要积极应对环境变化，巩固“三去一降一补”成果，着力构建高层次、高技术、高附加值、高外向度和具有综合竞争优势的现代产业体系。

坚定民营经济开拓发展信心。加强政策宣传，强化精准落实。做实企业服务中心。在项目准入、资金申请等方面支持民营企业创新发展。大力落实减税降费政策。利用好政银担、税融通、续贷过桥等政策，引导产业基金投向民营企业，用好省级民营企业纾困救助基金。完善政企沟通长效机制，保护企业家合法权益，构建民营企业公平竞争环境。

坚定传统产业转型发展决心。围绕主导产业抓技改，实施技改“双百”工程。实施企业兼并重组项目20个，加快淘汰落后产能。推动汽车产业向智能化、电动化、网联化方向发展，推动材料产业向节能环保方向转变，推动电器产业向智能家居延伸，增强电缆产业自主创新能力与市场竞争力。深入推进军民融合发展。支持奇瑞、海螺、新兴铸管等龙头企业发展高端制造、智能制造、绿色制造和服务型制造。培育智能工厂、数字化车间，完成“企业登云”700家以上。开展工业增品种、提品质、创品牌“三品”行动，打造一批“单打冠军”。

坚定新兴产业创新发展恒心。把握产业变革趋势，加快突破核心技术，大力推动“三重一创”建设。立足产业前沿，聚焦培育机器人、新能源汽车、航空、3D打印、快递物流智能装备等特色优势产业。建设国内领先的研发创新平台，支撑产业发展。

坚定现代服务业集聚发展雄心。制定加快现代服务业高质量发展的实施意见。重点发展现代物流、金融、新型信息服务、工业设计及检验检测等现代生产性服务业，新型商贸、文化旅游、健康养老、教育培训等现代生活性服务业。力争新增省级服务业集聚区1家、省级服务业集聚区示范园区1家。积极参与科创板块试点，持续推进企业对接多层次资本市场，力争2家企业首发上市，完成直接融资400亿元。加快旅游基础设施和公共服务体系建设，加快城东文化旅游集聚发展。

坚定三次产业融合发展初心。建设数字芜湖，培育数字经济新动能，支持芜湖电信扩建云中心二期，打造“安徽数谷”，规划建设大数据产业园，推动数字产业化、产业数字化，打造资源型、技术型、融合型、服务型数字经济。扎实推进乡村振兴战略，加快田园综合体建设，支持无为县、芜湖县六郎镇三次产业融合试点。以农业供给侧结构性改革为主线，优化农业区域布局，加快省级以上现代农业示范区转型升级。大力培育新型农业经营主体，鼓励发展高科技设施农业、休闲农业与乡村旅游，发展绿色农业和优质特色农产品。加快“芜湖大米”品牌培育工程建设。通过土地流转、入股、托管等多种形式，提高农业产业化经营水平。深化农村改革，实现农村“三变”改革镇级全覆盖。提升国省干道公路等级，推进农村公路扩面延伸工程。

（三）全力抓创新，集聚未来潜能。

自主创新是开放环境下的创新，“不拒众流，方为江海”。我们要站在区域视角、囊括全国视线、具备全球视野，促进创新环境优化、氛围浓厚，提升产业链水平。

建好科创载体平台。呼应合肥综合性国家科学中心建设，统筹推进国家自主创新示范区和国家创新型城市建设，支持重大公共研发平台和高水平研发机构发展，加快建设省技术创新中心，加强重大公共研发平台绩效考核。加快高新技术企业培育，壮大高新技术产业。启动市产业创新馆和西电芜湖研究院、中科大智慧城市研究院建设。强化知识产权创造保护运用，组建产业技术研究院等新型研发机构，鼓励企业研发机构提升创新能力。组建航空、现代农机等战略性新兴产业知识产权联盟。

用好区域创新展台。发挥G60科创走廊、合肥都市圈、南京都市圈区域合作和人才共享的叠加优势，积极利用区域创新资源，构建合作共赢的伙伴关系。深化人才发展体制机制改革，鼓励企业事业单位建立高校、科研院所实践基地，鼓励高校、科研院所、公立医院等事业单位聘用高层次人才。加快培育重点行业、重要领域人才，畅通人才流动机制，优化高层次人才“一站式”服务。

搭好成果转化舞台。创新产学研合作机制，建立科技成果信息共享与发布体系，举办大院大所合作暨科技成果对接会、全国机器人专利大赛等创新创业赛事。完善科技创新创业服务机制，支持高新技术创业服务中心、皖江技术转移转化中心、皖江知识产权运营中心、科智汇科技成果路演中心等科技创新服务机构发展。实施一批市级科技计划项目，促进科技成果转化。

（四）大力打基础，完善城市功能。

我们要找准“长江经济带大城市、皖南片中心城市”的城市定位，推动长三角地区更高质量一体化发展，坚持拥江发展、联动发展，谋求最优方案，提升城市治理水平。

区域交通提标扩容。加快建设全国综合交通枢纽、打造长江经济带立体交通走廊重要节点。开工建设机场快速路，芜合、芜马、芜宣高速公路改扩建，峨山路东延伸、大茆立交等重点工程。加快推进城市轨道交通、城区快速路网体系等工程建设。建成商合杭高铁大桥、皖赣铁路新建双线、芜湖宣城机场飞行区主体工程。打造智慧城市、公交都市，建成超算中心。对接毗邻城市，提升交通基础设施的互联互通水平。

市政设施提档升级。推进“厕所革命”和滨江公园二期、人民公园一期、汀棠公园人行过街天桥等项目。完成朱家桥等4座污水处理厂提标改造，开工建设城南等4座污水处理厂，完善市区污水主干网建设，推进老旧小区及公共单位雨污分流改造、江北供水供气污水设施建设。完成分流制排水系统雨污混接排查和整治工作。推进长江芜湖段治理、芜当联圩加固提升、青弋江芜湖闸枢纽工程前期工作。完成青弋江、裕溪河等支流治理，启动凤凰颈新站等大型排涝泵站建设。

城市风貌提色增彩。凸显芜湖中西交融的文化底蕴，加快青安江生态修复工程、“十里江湾”景观带建设。推进城市建设与旅游融合发展。继续开展贴近群众“六走进”行动，提升城乡管理和文明创建水平。实施百年历史建筑复活计划，以范罗山、雨耕山等为核心打造开埠文化商业区，加快芜湖古城建设。

（五）聚力促开放，释放改革动能。

芜湖“坐拥长江水，通向太平洋”。我们要传承开放之先、引领风气之先，从长江经济带、全国乃至全球等多维度审视自身，统筹发挥综合优势，增强微观主体活力，畅通区域经济循环。

自身找准定位。抢抓“十个一”融合机遇：一“角”就是长三角，更高质量一体化发展已上升为国家战略，办好区域协作重要会议；一“廊”就是G60科创走廊，九城协同创新；一“带”就是长江经济带，建设“共抓大保护、不搞大开发”绿色发展示范区；一“港”就是芜湖港，借力全省港口一体化、港航协同化发展的优势，做大做强港口经济；一“会”就是科博会，今年要办好第九届，在提升档次、创优品牌、扩大影响上聚力用劲；一“节”就是动漫节，办好第六届，打响欢乐芜湖品牌；一“赛”就是机器人大赛，以大赛聚人才、兴产业；一“园”就是雕塑公园，巩固发展中国第一、世界一流的成果；一“台”就是上海虹桥商务区城市展示平台，打造招商引资平台、通往国际窗口和品牌发布平台；一“群”就是长三角世界级机场群，突出特色，错位发展。

改革精准到位。加快国资国企改革，加强与央企的合作发展。敢于先行先试，深化“一企一组一策”帮扶机制，推进“四送一服”双千工程，打造“四最”营商环境，扶持实体经济发展。深化中小企业信用贷试点。引导企业调整进出口结构，开拓国际市场。探索投资项目承诺制改革。加快推进“放管服”改革、政务服务“一网、一门、一次”改革，确保政务服务中心事项进驻率提升到90%以上。全面推开证照分离改革，深化电子证照推广应用，建设电子认证统一管理云服务平台，实现“一人一证、一证多用、一证通用”。

开放瞄准前位。复制推广自贸区第四批改革试点经验，积极申创中国（安徽）自贸区芜湖片区。全面落实外商投资企业的准入后国民待遇，探索重点外商投资企业“服务大使”制度，优化外商投资环境。提升贸易便利化水平，发挥进境指定口岸扩大进口作用，加快三山港对外开放进程，推广国际贸易“单一窗口”模式。支持奇瑞、海螺等企业走进“一带一路”。鼓励龙头企业开展跨国并购，提升核心竞争力。

（六）倾力惠民生，体现共享效能。

习近平总书记强调，要坚持以人民为中心的发展思想。我们要按照普惠性、保基本、均等化、可持续的要求，继续实施33项民生工程，提高共建共治共享水平。

脱贫为要。强化精准导向，提高脱贫质量，再减少贫困人口6000人以上。安排财政专项扶贫资金3.6亿元，推进扶贫九大工程，加大特殊贫困群体脱贫攻坚力度，稳固脱贫成效。研究解决那些收入水平略高于建档立卡贫困户群体缺乏政策支持等新问题。规范建设就业扶贫驿站、车间、基地。

教育为基。完善新一轮义务教育、学前教育布局规划。实施第三期学前教育行动计划，开展城镇小区配套幼儿园专项整治，加快公办园建设。统筹推进义务教育优质均衡发展，推进城市新区中小学校建设，化解部分区域上学难和“大班额”问题。发展现代职业教育，发挥芜湖市职业教育联盟作用，深化产教融合、校企合作。推进教育信息化2.0行动计划，建设智慧校园，推动公益“阳光云课”应用，构建线下与线上相结合的教育新生态。推进“三名工程”建设，打造优质教师队伍。扩大特殊教育资源供给。加强普通高中课程建设。支持在芜高校发展。

就业为先。完善稳就业普惠式政策体系。落实退役军人安置和帮扶就业政策。建设皖江人力资源公共职业训练基地，打造技工强市。构建以第三代社会保障卡为载体的“多卡融合、一卡通用”的“城市一卡通”应用模式。拓展增长空间和途径，确保居民收入与经济增长同步。完善社会保障体系，构建和谐劳动关系，让困难群众及时得到救助。扩大住房公积金政策覆盖面。加快构建房地产市场健康发展长效机制。

健康为本。持续深化医改，构建整合型城乡医疗服务体系，推进分级诊疗。探索开放居民电子健康档案。全面建立现代医院管理制度，加强综合监管。完成市妇幼保健院整体搬迁和市一院儿科项目建设，推进中医传承与创新研究中心建设。加快医疗卫生人才和全科医生队伍建设。加强重大疾病防控和健康教育。实施妇女儿童健康提升项目。争创国家卫生城市。推进医养结合试点，加快发展残疾人事业。

文化为源。推动文化事业和文化产业发展，不断提升文化软实力。加快文学艺术、新闻出版、广播电视、档案、地方志等事业发展。完善公共文化、体育设施，提高公共文化服务质量和水平。培育公益慈善品牌，扩大慈善救助范围。做好民族、宗教、侨务、外事、对台、科普、经济普查等工作。支持群团组织、社会组织履行职责。

安全为重。防范化解企业债务和房地产风险，严格政府债务管理，守住不发生区域性金融风险的底线。落实安全生产“党政同责、一岗双责、齐抓共管、失职追责”制度，加快建设农资农产品、食品药品等重要产品追溯体系。加强交通、消防、危化品、非煤矿山等重点行业领域安全生产专项整治。发展人防、气象、防灾减灾等各项事业。夯实信访基层基础工作，化解信访突出问题。完善重大事件应急处置。健全立体化信息化社会治安防控体系，严厉打击各类违法犯罪活动，坚持黑恶势力和“保护伞”一起打。加强双拥共建，支持驻芜部队建设，完善国防动员体系。

（七）努力强自身，转变政府职能。

政府干部是人民公仆。我们要不忘初心、牢记使命，以政府系统党的建设带动政府自身建设、带动城市发展，建设人民满意的法治政府、创新政府、廉洁政府和服务型政府。

坚持党的领导。落实全面从严治党要求，以党的政治建设为统领，树牢“四个意识”，坚定“四个自信”，坚决做到“两个维护”。扎实做好上级巡视反馈问题整改。注重方法论上的深度学、深刻思、深层悟、深情用，推动政府系统党组中心组学习规范化、机制化、常态化、应用化。

坚持把纪律和规矩挺在前面。持之以恒贯彻中央八项规定和省、市转变工作作风的规定精神，坚决防止“四风”问题反弹回潮。加强廉政重点领域监管，织密扎牢制度笼子。加强审计监督。加大群众身边腐败问题的整治力度。加强纪律教育，让政府工作人员知敬畏、存戒惧、守底线。

坚持依法行政。落实法治政府建设实施纲要，严格执行重大行政决策程序规定，加强重大事项合法性审查，切实提升科学决策、民主决策、依法决策水平。完善权责清单体系，以制度刚性约束权力任性。加强行政执法队伍建设，严格规范公正文明执法。自觉接受人大监督、政协民主监督、监察机关监督、社会舆论和人民群众监督。

坚持担当作为。聚焦建设忠诚干净担当的高素质干部队伍要求，努力增强本领，激励政府工作人员新时代新担当新作为。树牢正确的政绩观，解放思想、放大格局，多做打基础、利长远的工作。改进和创新工作方法，注重实践论上的抓关系、找联系、善叠加、会穿透，统筹协调抓好各项工作。

坚持优化服务。实施市县区机构改革。深化“亲切服务”，推进机关效能建设，不断提升群众办事满意度。关心民营企业发展、民营企业家成长，构建亲清新型政商关系。全面推进政务公开，让人民监督政府行为，让权力在阳光下运行。

各位代表！

新时代鼓舞人心，新征程催人奋进。我们要更加紧密地团结在以习近平同志为核心的党中央周围，在中共芜湖市委的坚强领导下，勇往直前，再创辉煌，以优异成绩庆祝中华人民共和国成立70周年！
